# Bébé pas comme les autres

**DOI:** 10.11604/pamj.2014.19.143.5535

**Published:** 2014-10-14

**Authors:** Nada El Moussaoui, Fatima Jabouirik

**Affiliations:** 1 Service de Dermatologie et Vénéréologie, CHU Ibn Sina, Université Mohamed V, Souissi, Rabat, Maroc; 2 Service de Pédiatrie, Hôpital d'enfant, Université Mohamed V, Souissi, Rabat, Maroc

**Keywords:** Bébé collodion, génodermatose, conseil génétique, collodion baby, genodermatosis, genetic counseling

## Image in medicine

Le bébé collodion est un aspect clinique grave caractérisé par une peau luisante, tendue et vernissée; une membrane rigide responsable d'un syndrome dysmorphique. Sur le plan génétique on note une anomalie de la kératinisation, le plus souvent de transmission autosomique récessive. Le diagnostic repose essentiellement sur les données cliniques et anamnestiques, mais peut être confirmé, dans certains cas, par des examens biologiques, histologiques et génétiques. Le traitement comprend dans tous les cas des soins locaux et des émollients. Un traitement systémique à base de rétinoïdes aromatiques per os peut être indiqué dans les formes sévères. L’évolution de l’état clinique d'un bébé collodion est variable en fonction de la pathologie de base. Le conseil génétique ainsi que le diagnostic anténatal jouent un rôle fondamental dans la prévention de ce type de génodermatose. Notre étude a porté sur 7 cas de bébés collodions, issus de parents consanguins. L’âge de nos malades variait entre J4 et 3 mois. Les filles étaient plus touchées que les garçons. Le diagnostic positif était posé seulement sur l'aspect clinique des bébés. Tous les bébés ont été mis sous soins locaux et émollients. Une antibiothérapie était préconisée en cas de surinfection.

**Figure 1:**
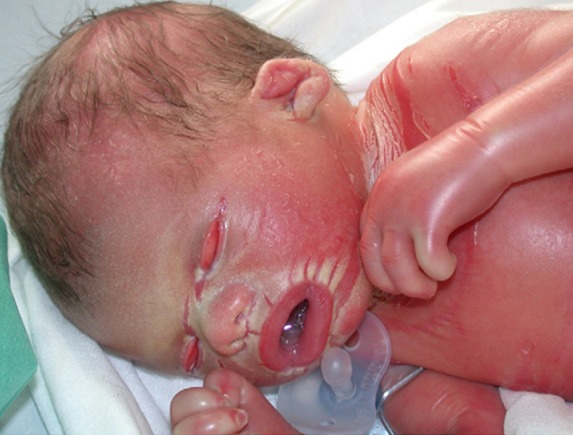
Un syndrome dysmorphique chez un bébé collodion

